# A novel assay of excess plasma kallikrein-kinin system activation in hereditary angioedema

**DOI:** 10.3389/falgy.2024.1436855

**Published:** 2024-09-17

**Authors:** Dan Sexton, Ryan Faucette, Melody Rivera-Hernandez, Jon A. Kenniston, Nikolaos Papaioannou, Janja Cosic, Kris Kopacz, Gary Salmon, Chantal Beauchemin, Salomé Juethner, Dave Yeung

**Affiliations:** ^1^ Takeda Development Center Americas Inc., Cambridge, MA, United States; ^2^ Charles River Laboratories, Harlow, United Kingdom; ^3^ Takeda Pharmaceuticals USA, Inc., Lexington, MA, United States

**Keywords:** biomarkers, phage display, bradykinin, plasma kallikrein, hereditary angioedema

## Abstract

**Background:**

Cleaved high-molecular-weight kininogen (HKa) is a disease state biomarker of kallikrein-kinin system (KKS) activation in patients with hereditary angioedema due to C1 inhibitor deficiency (HAE-C1INH), the endogenous inhibitor of plasma kallikrein (PKa).

**Objective:**

Develop an HKa-specific enzyme-linked immunosorbent assay (ELISA) to monitor KKS activation in the plasma of HAE-C1INH patients.

**Methods:**

A novel HKa-specific antibody was discovered by antibody phage display and used as a capture reagent to develop an HKa-specific ELISA.

**Results:**

Specific HKa detection following KKS activation was observed in plasma from healthy controls but not in prekallikrein-, high-molecular-weight kininogen-, or coagulation factor XII (FXII)-deficient plasma. HKa levels in plasma collected from HAE-C1INH patients in a disease quiescent state were higher than in plasma from healthy controls and increased further in HAE-C1INH plasma collected during an angioedema attack. The specificity of the assay for PKa-mediated HKa generation in minimally diluted plasma activated with exogenous FXIIa was demonstrated using a specific monoclonal antibody inhibitor (lanadelumab, IC_50_ = 0.044 µM).

**Conclusions:**

An ELISA was developed for the specific and quantitative detection of HKa in human plasma to support HAE-C1INH drug development. Improved quantification of the HKa biomarker may facilitate further pathophysiologic insight into HAE-C1INH and other diseases mediated by a dysregulated KKS and may enable the design of highly potent inhibitors targeting this pathway.

## 1 Introduction

The plasma kallikrein-kinin system (KKS) lies at an interface between *coagulation* through the activation of the intrinsic coagulation pathway, and *inflammation* via its ability to generate bradykinin, a potent mediator of vascular permeability, inflammation, and pain (1). The KKS consists of prekallikrein (PK), high-molecular-weight kininogen (HK), and coagulation factor XII (FXII) and is activated by FXII autoactivation upon exposure to negatively charged surfaces, the exact endogenous identity of which remains elusive (2). KKS activation can also involve membrane-associated prolylcarboxypeptidase or a complex of PK, HK, and heat shock protein 90 (HSP90) on endothelial cells (2).

The initial generation of plasma kallikrein (PKa) promotes a rapid burst of localized KKS activation through the PKa-catalyzed conversion of FXII to its active form (FXIIa), which generates additional PKa (3). FXIIa can initiate the intrinsic coagulation pathway through the generation of FXIa, and inhibitors of FXIIa and FXIa have been proposed as novel anti-thrombotic agents, as they have the potential to be anti-thrombotic without increasing the risk of bleeding (4). PKa also cleaves HK to liberate bradykinin, a 9-amino acid peptide, and cleaved HK (HKa). Bradykinin-mediated edema occurs through the activation of the constitutively expressed B2 receptor or the inducible B1 receptor (5). Endogenous KKS activation is regulated through protease inhibitors, including *α*2-macroglobulin and C1 inhibitor (C1INH) (6).

A genetic deficiency in C1INH leads to dysregulated KKS activation, excess bradykinin production, and the debilitating disease of hereditary angioedema due to C1INH deficiency (HAE-C1INH) (3). HAE-C1INH is an autosomal dominant disease manifesting as intermittent edematous attacks at locations that include the gastrointestinal tract, facial tissue, the upper airway, oropharynx, urogenital region, and/or extremities. Patients with HAE-C1INH type I have mutations in the *SERPING1* gene that lead to a deficiency in the total amount of C1INH protein, whereas HAE-C1INH type II patients have a dysfunctional C1INH (7). The role of excess KKS activation in HAE-C1INH pathophysiology has been validated by the approval of therapies that include C1INH protein replacement products, a bradykinin B2 receptor antagonist (icatibant), and PKa inhibitors (ecallantide, lanadelumab, and berotralstat).

HKa has previously been shown to be a disease state biomarker of KKS activation and is elevated in plasma from HAE-C1INH patients and other disease states (8–18). We describe the development of a novel enzyme-linked immunosorbent assay (ELISA) to specifically detect HKa in human plasma and demonstrate the utility of the assay in comparing the *in vitro* potency of PKa inhibitors.

## 2 Methods

### 2.1 Materials

HK, HKa, PKa, FXII, and FXIIa were obtained from Enzyme Research Laboratories (South Bend, IN, USA). Low-molecular-weight kininogen (LK) was procured from Athens Research (Athens, GA, USA). Mouse monoclonal antibodies 11H05 and 13B12 were generated at BBI Solutions (Portland, ME, USA) following immunization with human HKa. PK-deficient and FXII-deficient plasma were obtained from George King Biomedical (Overland Park, KS, USA) using sodium citrate as an anticoagulant. HK-deficient human plasma using sodium citrate as an anticoagulant was obtained from Affinity Biologicals (Ancaster, ON, Canada). SCAT169 plastic evacuated blood collection tubes (5 ml volume) were prepared by Prolytix (Essex Junction, VT, USA) and contained 0.5 ml of a 10× concentrated mixture [100 mM benzamidine, 400 µg/ml polybrene, 2 mg/ml soybean trypsin inhibitor, 20 mM EDTA, 263 µM leupeptin, and 20 mM 4-(2-aminoethyl) benzenesulfonyl fluoride hydrochloride (AEBSF) dissolved in acid citrate dextrose (100 mM sodium citrate, 67 mM citric acid, and 2% dextrose, pH 4.5)]. P100 Blood Collection System tubes were purchased from BD Biosciences (San Jose, CA, USA). Normal human plasma, collected with sodium citrate, SCAT169, or P100 tubes, was obtained from BioIVT (Westbury, NY, USA). Ellagic acid was obtained as a 0.003% solution (Pacific Hemostasis APTT-XL reagent) from Thermo Fisher Scientific (Waltham, MA, USA).

### 2.2 Plasma collection

Plasma was collected from healthy volunteers (HVs) or patients with HAE-C1INH with approval by the institutional review boards or ethics committees of the participating institutions (see Acknowledgments). Plasma was collected from HAE-C1INH patients at baseline (e.g., not during an attack) or within 6 h of the onset of an angioedema attack. HAE-C1INH plasma was collected and analyzed before the availability of recently approved prophylactic therapies (e.g., lanadelumab, berotralstat, or subcutaneous C1INH). All healthy volunteers and patients provided written informed consent to use the blood for the investigation of exploratory biomarkers of KKS activation. To minimize the *ex vivo* activation of the KKS during blood collection, plasma was collected from subjects with HAE-C1INH and healthy controls by means of a clean venipuncture with a butterfly needle/catheter kit (BD Biosciences, #367296). The first tube of blood was discarded, and blood was collected into polypropylene-evacuated tubes containing either 3.2% sodium citrate (BD Biosciences, San Jose, CA, USA) or a protease inhibitor/anticoagulant mixture (SCAT169 or P100 tubes). Blood samples were centrifuged within 1 h, and plasma was aliquoted and stored at −80°C until processing.

### 2.3 Phage display

Antibodies specific for HKa were discovered using a human antibody phage display library (19) by first performing a negative selection of the library with an input of approximately 1 × 10^12^ phage against biotinylated HK that was immobilized onto streptavidin-coated magnetic beads (Dynabeads™ M-280, Thermo Fisher Scientific). The depleted library was then incubated with biotinylated HKa immobilized on streptavidin-coated magnetic beads. The beads were extensively washed with phosphate buffered saline (PBS) buffer and used to infect *Escherichia coli* for phage output amplification to complete a round of selection. Three rounds of selection were performed before screening individual phage colonies using an ELISA with biotinylated HK and HKa immobilized on streptavidin-coated plates followed by detection with horseradish peroxidase (HRP)-conjugated anti-M13 antibody and absorbance detection through substrate hydrolysis of 3,3′,5,5′-tetramethylbenzidine (TMB). Recombinant fragment antigen-binding (Fab) fragments were expressed in *E. coli* and purified by protein A Sepharose (GE Healthcare, Piscataway, NJ, USA) (20). Recombinant immunoglobulin G (IgG1) human anti-HKa antibodies were transiently expressed in 293T cells and purified by protein A Sepharose chromatography. Fab specificity was determined by coating 96-well or 384-well plates with each purified Fab and measuring the binding to biotinylated HK, biotinylated HKa, or biotinylated LK.

### 2.4 Western blot

Plasma was analyzed by a western blot assay (WBA) after activation with various agents (e.g., ellagic acid, FXIIa, or PKa) or after the addition of a 1/10th volume of the 10× SCAT169 cocktail upon thawing of frozen plasma (45 µl) and diluted (1:20) in Tris-buffered saline. Samples were further diluted in NuPAGE® Sample Buffer (Life Technologies Corporation, Carlsbad, CA, USA) containing 50 mM of dithiothreitol and analyzed by sodium dodecyl sulfate-polyacrylamide gel electrophoresis using a precast 4%–12% acrylamide gradient gel and the 2-(N-morpholino) ethanesulfonic acid sodium dodecyl sulfate buffer system (Life Technologies Corporation). Protein was transferred from the gel to a nitrocellulose membrane using the iBlot® system (Life Technologies Corporation). Membranes were blocked in Odyssey blocking buffer (LI-COR Biosciences, Lincoln, NE, USA) containing 0.2% Tween 20 and incubated for 1 h at room temperature (RT) with a mouse monoclonal antibody that specifically binds the light chain of HK (Clone 11H05) diluted to 1 µg/ml in Odyssey blocking buffer. Goat anti-mouse IgG IRDye 680 was prepared at a 1:15,000 dilution (LI-COR Biosciences) and the membrane was incubated for 1 h at RT. After a PBS wash, membranes were analyzed using a LI-COR Odyssey CLx (LI-COR Biosciences). The percentage of HKa was calculated from the fluorescent intensities of bands attributed to the cleaved light chain of HKa compared with total HK in each sample.

### 2.5 HKa ELISA

For the initial ELISA, the Fab version of the HKa-specific antibody (M4-B4 Fab) was coated on 96-well plates (Costar, #9018) overnight in PBS before being washed and blocked with 2% bovine serum albumin (BSA) buffer. Samples, standards, and quality controls were diluted in LowCross-Buffer (Boca Scientific, Boca Raton, FL, USA) and added to the plate. After an incubation and wash, plate-bound HKa was detected by adding HRP-labeled sheep anti-HK polyclonal antibody (Affinity Biologicals, Ancaster, ON, Canada). After incubation with the detection antibody, the plate was washed, TMB substrate was added, the incubation was stopped with phosphoric acid, and the optical density was measured at 450 nm with subtraction at 630 nm. Further ELISA optimization involved coating the M4-B4 Fab or IgG on Nunc MaxiSorp plates overnight in PBS, before washing and blocking with BSA (protease/IgG-free) buffer. Samples, standards, and quality controls diluted in 0.1% BSA buffer were added to the plate and, after a subsequent incubation, plate-bound HKa was detected by adding mouse anti-HK monoclonal antibodies (either pooled 11H05 and 13B12 or just 11H05), which were selected based on their ability to form a sandwich pair with the M4-B4. The plate was washed and a secondary goat anti-mouse IgG HRP was added to the plate, followed by TMB substrate, stopping with phosphoric acid, and OD measurement at 450 nm with subtraction at 630 nm.

### 2.6 *Ex vivo* KKS activation assay in minimally diluted plasma

To assess the inhibition potency of PKa inhibitors, 2.5 µl of serially diluted inhibitors (synthetic compounds were diluted in DMSO and biologics were diluted in PBS) were dispensed into 96-well PCR plates (Thermo Fisher Scientific, #AB0600) followed by the addition of 45 µl of sodium citrate plasma (pooled from three donors). Samples were incubated for 5 min at RT before the addition of 2.5 µl of 100 nM human FXIIa solution followed by incubation in a shallow 37°C water bath for 30 min. The activation was terminated by a 150-fold dilution of plasma samples in ice-cold dilution buffer (1% BSA/0.01% casein in PBS).

### 2.7 Statistical methods

Western blot and ELISA data comparisons for HKa levels in plasma from healthy volunteers or patients with HAE-C1INH were analyzed using the Mann–Whitney *U*-test (SigmaPlot software, Grafiti LLC, Palo Alto, CA, USA), for which *p* < 0.05 was considered significant. Receiver operator characteristic (ROC) analysis was performed using Prism software (GraphPad, Boston, MA, USA). ELISA standard curves were fitted to a four-parameter logistic equation (Softmax, San Jose, CA, USA). Half-maximal inhibitory concentration (IC_50_) values were obtained through a non-linear regression analysis of dose responses fitted to averaged values from at least three experiments of duplicate measurements through non-linear regression (Prism software) using a three-parameter hyperbolic competitive equation {Y = Bottom + [Top-Bottom]/[1 + (X/IC_50_)]} to provide estimated IC_50_ values.

## 3 Results

**Figure 1:**
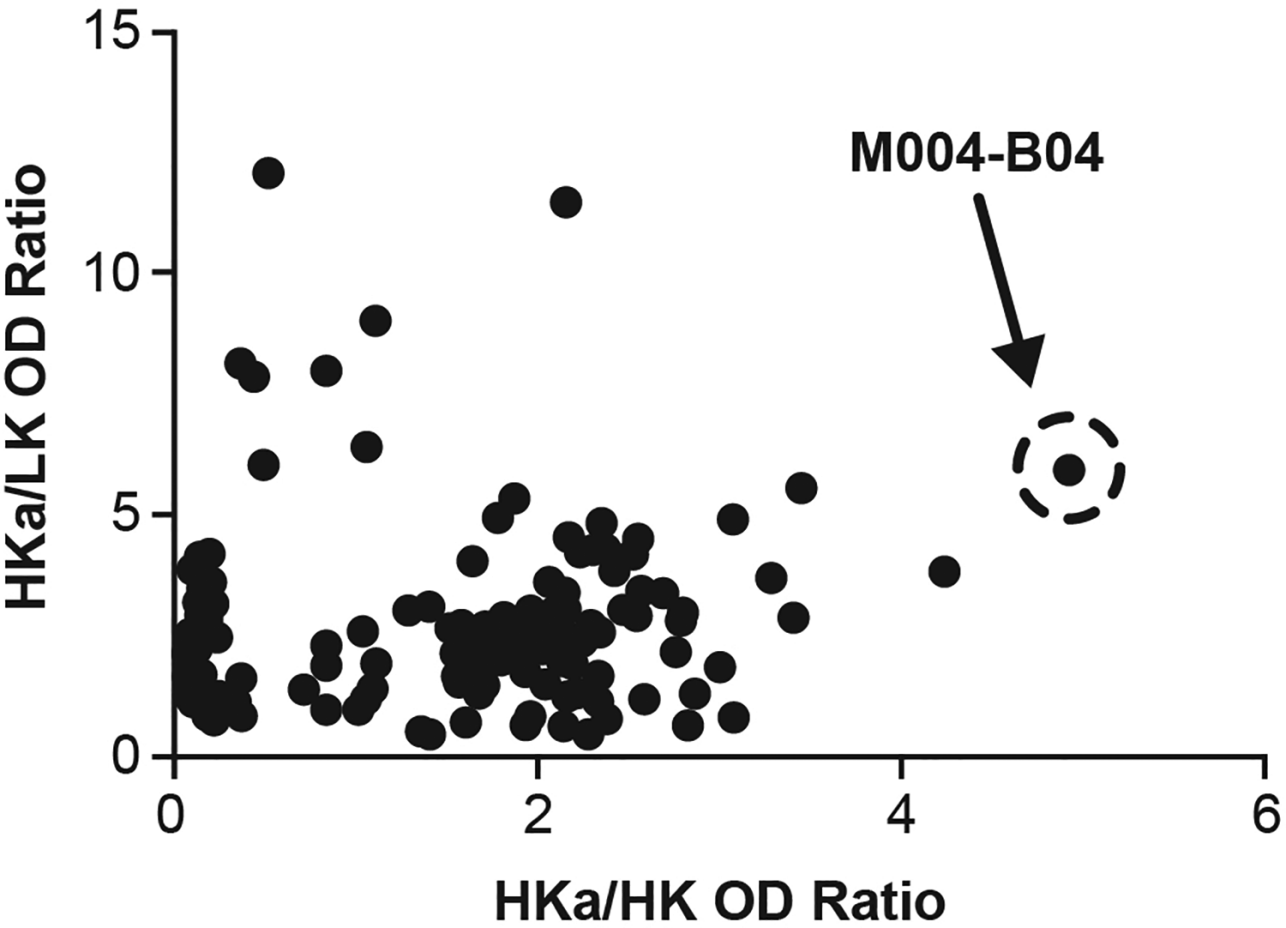
Discovery of an HKa-specific antibody by phage display selection and screening. Recombinant Fab fragments were passively immobilized onto 384-well plates before the addition of either biotinylated HKa, intact HK, or LK followed by detection using streptavidin-HRP and TMB as described in the Methods section. Fab, fragment antigen-binding; HK, high-molecular-weight kininogen; HKa, cleaved high-molecular-weight kininogen; HRP, horseradish peroxidase; LK, low-molecular-weight kininogen; OD, optical density; TMB, 3,3′,5,5′-tetramethylbenzidine.

A human antibody phage display library (19) was depleted against HK and panned against HKa, from which over 6,700 putative positive phage colonies were obtained. Phage clones positive for binding to immobilized HKa were selected, sub-cloned for soluble Fab fragment expression, and further screened for HKa specificity by an ELISA. As target immobilization could mask epitope(s) present in HKa as opposed to HK, and as the goal was to develop an ELISA to detect soluble HKa in plasma, subsequent screening was performed using purified Fabs immobilized on 384-well or 96-well plates with the detection of either biotinylated HK, LK, or HKa binding via streptavidin-HRP. The specificity of 190 anti-kininogen binding Fabs is shown in Figure 1 where the Fab M4-B4 (aka M004-B04) exhibited the highest specificity for HKa vs. HK or LK (see Supplementary Figure S1 for the specificity of two other Fabs and Supplementary Figure S8 for the amino acid sequence of the M4-B4 Fab).

**Figure 2:**
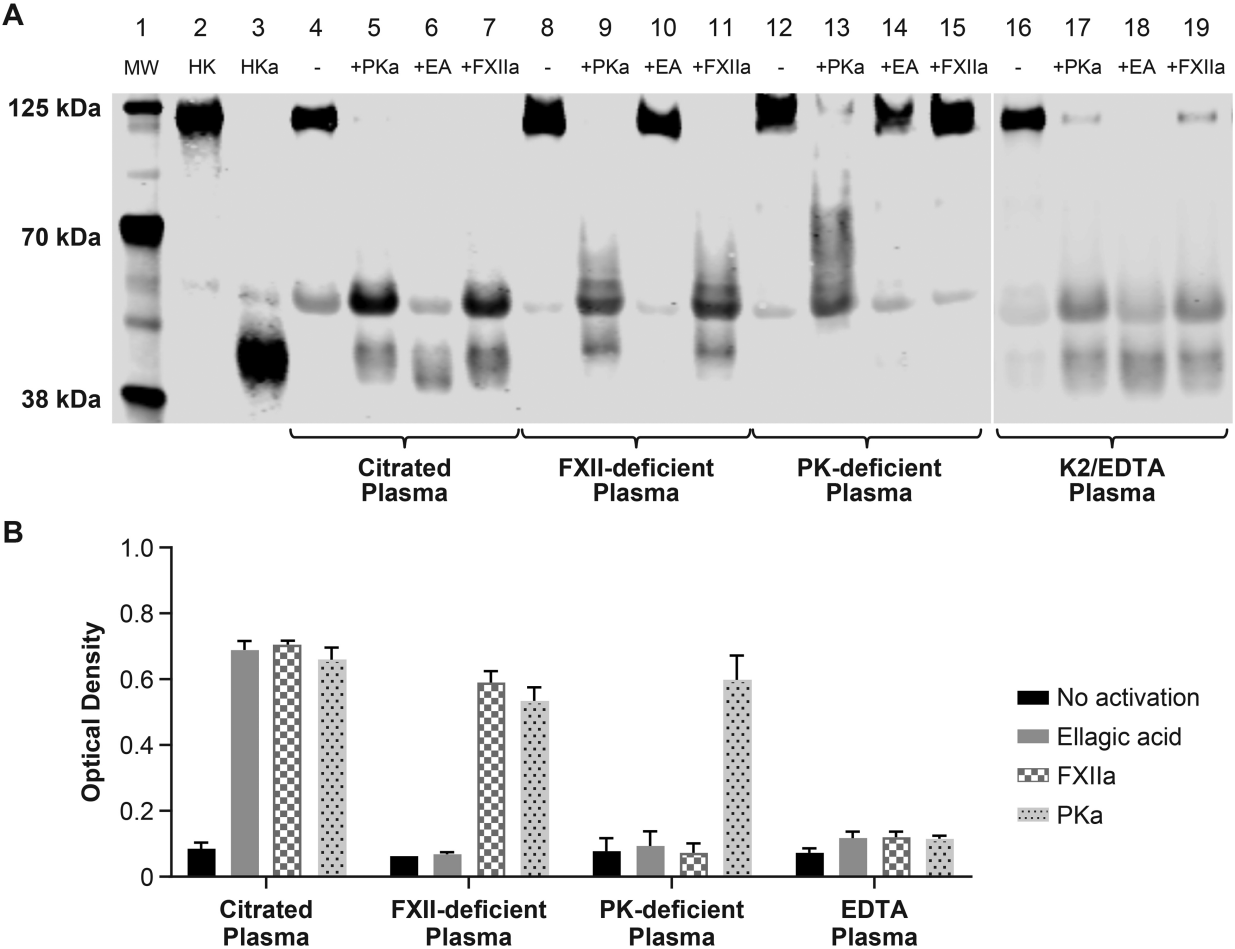
Characterization of M4-B4 reactivity in human plasma. Sodium citrate and EDTA plasma from healthy volunteers, FXII-deficient sodium citrate plasma, and PK-deficient sodium citrate plasma were either untreated or activated for 20 min at 37°C with either 100 nM PKa, ellagic acid reagent, or 10 nM FXIIa before analysis by western blotting **(A)** using an anti-HK monoclonal antibody (11H05) or by an ELISA **(B)** using M4-B4 as a capture antibody and a 1:1 mixture of 11H05 and 13B12 anti-HK monoclonal antibodies as detection antibodies. Lane composition are as follows: molecular-weight markers (MW) (lane 1), purified HK (lane 2), purified HKa (lane 3), citrate plasma (lane 4), citrate plasma + PKa (lane 5), citrate plasma + ellagic acid (lane 6), citrate plasma + FXIIa (lane 7), FXII-deficient plasma (lane 8), FXII-deficient plasma + PKa (lane 9), FXII-deficient plasma + ellagic acid (lane 10), FXII-deficient plasma + FXIIa (lane 11), prekallikrein-deficient plasma (lane 12), prekallikrein-deficient plasma + PKa (lane 13), prekallikrein-deficient plasma + ellagic acid (lane 14), prekallikrein-deficient plasma + FXIIa (lane 15), EDTA plasma (lane 16), EDTA-deficient plasma + PKa (lane 17), EDTA-deficient plasma + ellagic acid (lane 18), and EDTA-deficient plasma + FXIIa (lane 19). EA, ellagic acid; FXIIa, coagulation factor XIIa; HK, high-molecular-weight kininogen; HKa, cleaved high-molecular-weight kininogen; K2/EDTA, dipotassium ethylenediaminetetraacetic acid; LK, low-molecular-weight kininogen; MW, molecular weight; PK, prekallikrein; PKa, plasma kallikrein.

The ability of the M4-B4 antibody to detect HKa was investigated in human plasma treated with the KKS activator ellagic acid (21), purified FXIIa, or purified PKa (Figure 2). KKS activation was assessed by WBA (Figure 2A) using a mouse monoclonal antibody (11H05) that binds intact HK as well as the 56- and 46-kDa forms of the HKa light chain. With WBA, activated human plasma showed a reduction in the band corresponding to intact HK and increases in the two bands corresponding to light chain (Figure 2A). Activated plasma displayed an increased HKa ELISA signal (Figure 2B). Of note, our ELISA did not detect endogenous HKa in plasma from cynomolgus monkeys, mice, or rats after activation with ellagic acid (data not shown).

FXII- and PK-deficient plasma demonstrated the specificity of M4-B4 for HKa. As expected, HKa was not generated in FXII-deficient plasma treated with ellagic acid (lane 10 of Figure 2A and the solid gray bar in Figure 2B), but was produced after the addition of FXIIa (lane 11 of Figure 2A and the hatched bar in Figure 2B) or PKa (lane 9 of Figure 2A and the dotted bar in Figure 2B). Ellagic acid activates the KKS through the formation of an insoluble metal ion complex that serves as a charged surface to promote FXII autoactivation to FXIIa (22). It was therefore expected that ellagic acid would be unable to activate the KKS in FXII-deficient or PK-deficient plasma. PK-deficient plasma was only activated by the addition of PKa.

Human plasma collected using EDTA as an anticoagulant was activated similarly to sodium citrate plasma, as shown by HKa generation using WBA (Figure 2A), supporting the previous observation that metal ions are not required for KKS activation (6). Interestingly, despite HKa formation in activated EDTA plasma by WBA (Figure 2B), HKa was not detected by an ELISA (Figure 2B), suggesting that M4-B4 binding to HKa may be metal ion-dependent, which was restored by the addition of zinc chloride at concentrations that exceeded the EDTA concentration in the blood collection tube (Supplementary Figure S2). Surface plasmon resonance analyses demonstrated that M4-B4 bound HKa with approximately a sixfold higher affinity in the presence of zinc chloride (K_D_ = 1.06 ± 0.25 nM) than in its absence (K_D_ = 6.08 ± 0.26 nM) (Supplementary Figure S3 and Supplementary Table S2). Zinc has been previously shown to induce conformational changes in HK (23, 24).

**Figure 3:**
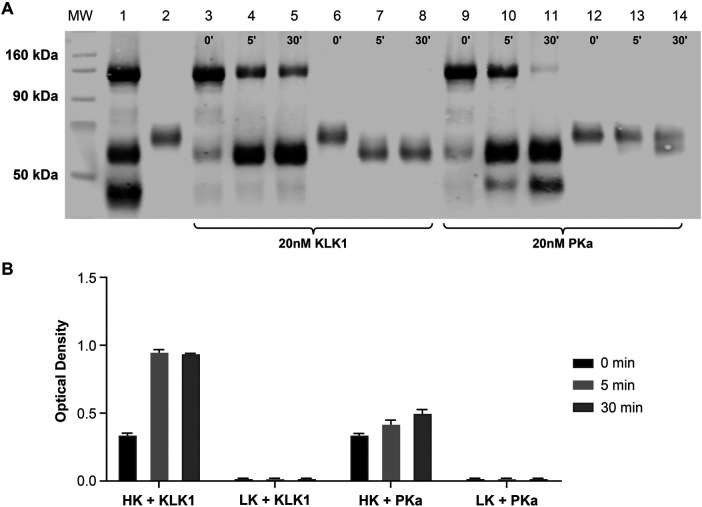
Comparative kininogen digestion by tissue vs. plasma kallikrein. Purified HK and LK were analyzed by western blotting **(A)** or an ELISA **(B)** using M4-B4 as a capture antibody and 11H05 and 13B12 as detection antibodies following incubation with either KLK1 or PKa. The western blot analysis was performed using a polyclonal sheep anti-kininogen antibody for detection that cross-reacted with LK as well as HK. The lane compositions are as follows: molecular-weight markers (MW), a mixture of purified intact HK and HKa (1.5 µM each) (lane 1); purified LK (1.5 µM) (lane 2); HK (1.5 µM) incubated with 20 nM KLK1 for 0, 5, and 30 min (lanes 3–5); LK (1.5 µM) incubated with 20 nM KLK1 for 0, 5, and 30 min (lanes 6–8); HK (1.5 µM) incubated with 20 nM PKa for 0, 5, and 30 min (lanes 9–11); and LK (1.5 µM) incubated with 20 nM PKa for 0, 5, and 30 min (lanes 12–14). HK, high-molecular-weight kininogen; HKa, cleaved high-molecular-weight kininogen; KLK1, tissue kallikrein 1; LK, low-molecular-weight kininogen; MW, molecular weight; PK, prekallikrein; PKa, plasma kallikrein.

HK and LK are cleaved by tissue kallikrein 1 (KLK1) (25), as confirmed by WBA (Figure 3A). Although the form of HKa generated by KLK1 was detected by the ELISA, KLK1-cleaved LK was not detected (Figure 3B). KLK1-cleaved HK consists mainly of the 56-kDa light chain (lanes 4 and 5 of Figure 3A), whereas the purified HKa standard protein mainly consists of the 46-kDa light chain (see lane 2 of Figure 3A), which suggests that M4-B4 binds HKa at either light chain variant.

**Figure 4:**
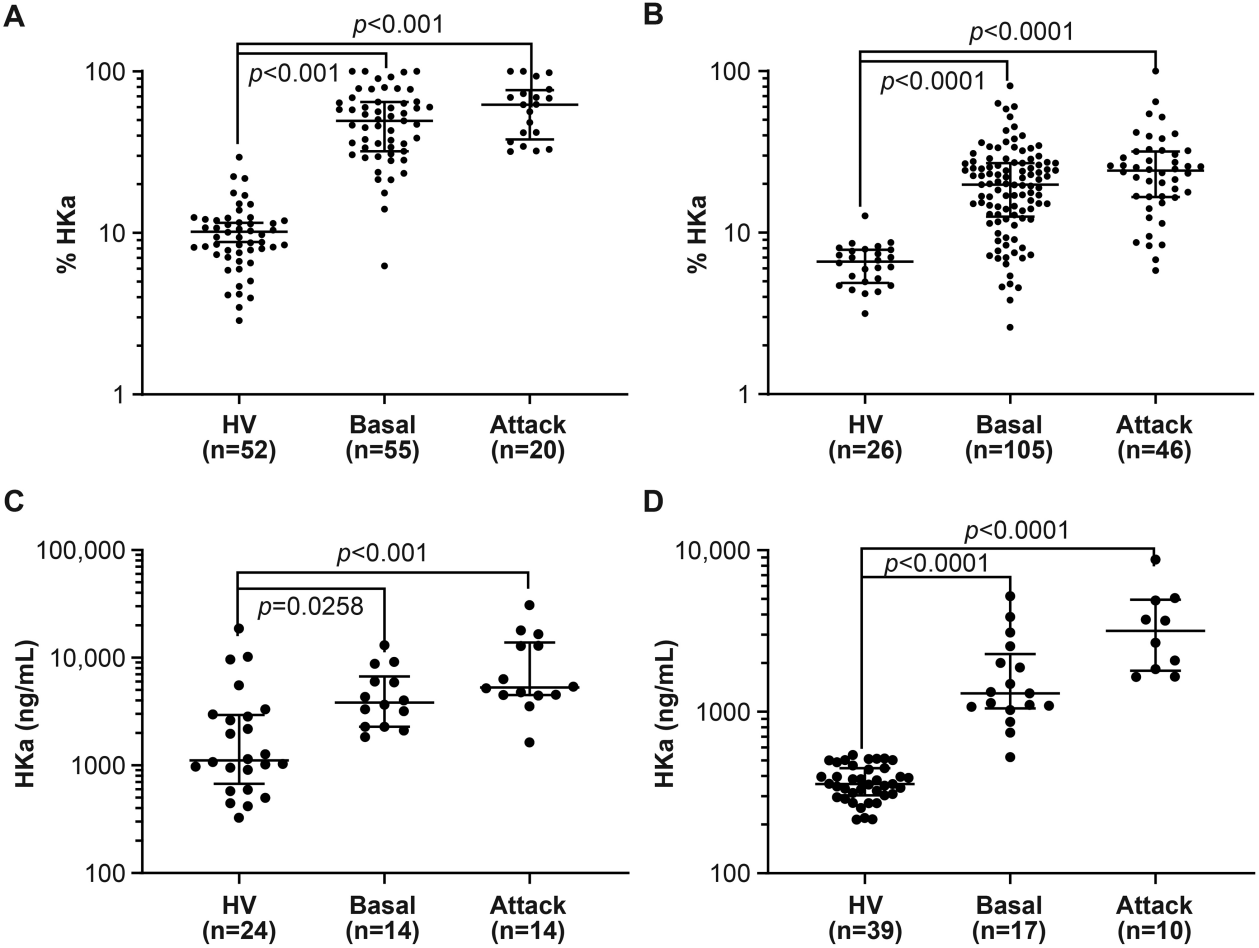
Comparison of HKa levels in healthy volunteers and HAE-C1INH patients by western blotting and an ELISA. The statistical analysis of the data in **(A–D)** is summarized in Supplementary Table S3. **(A)** Comparison of the percentage of HKa measured by western blotting in the citrated plasma of healthy volunteers with that from HAE-C1INH patients collected between (basal) and during an attack. **(B)** Comparison of the percentage of HKa measured by western blotting in the SCAT169 plasma of healthy volunteers with that from HAE-C1INH patients collected between (basal) and during an attack. **(C)** Comparison of HKa levels measured by an ELISA in the citrated plasma of healthy volunteers with those from HAE-C1INH patients collected between (basal) and during an attack. **(D)** Comparison of HKa levels measured by an ELISA in the SCAT169 plasma of healthy volunteers with those from HAE-C1INH patients collected between (basal) and during an attack. Group means and 95% confidence intervals are shown. Groups were compared with each other by one-way ANOVA. HAE-C1INH, hereditary angioedema due to a deficiency in total (type I) or functional C1 inhibitor protein (type II); HKa, cleaved high-molecular-weight kininogen; HV, healthy volunteer.

Standard curves prepared by spiking HKa into citrated plasma from HVs demonstrated a lower limit of quantitation of approximately 156 ng/ml at a dilution of 1:320 (Supplementary Figure S4). HKa levels in citrated and SCAT169 plasma from HVs and HAE-C1INH patients were compared using a WBA and an ELISA (Figure 4, Supplementary Table S1). HKa levels were higher (*p *< 0.05) in both plasma types from HAE-C1INH patients collected either during an attack or in a basal state of disease quiescence than from HVs, using either assay (Supplementary Table S3). Although the percentage of HKa by WBA was higher during an attack than under the basal disease state, it did not reach statistical significance (*p *< 0.05). In contrast, a statistical difference in HKa plasma levels between the basal and attack disease states was observed by an ELISA.

ROC analysis of WBA data demonstrated that HKa levels in citrated plasma can differentiate HAE-C1INH from HVs with an area under the curve (AUC) value of 0.977 for the comparison of basal with HVs or 1.0 for the comparison of attack with HVs (Supplementary Table S3, Supplementary Figure S5). Plasma HKa levels for HAE-C1INH patients in citrated plasma collected between attacks (i.e., basal samples) were less differentiated from attack samples (AUC = 0.625). Similarly, ROC analysis of WBA data demonstrated that HKa levels in SCAT169 plasma differentiates HAE-C1INH from HVs, with an AUC value of 0.915 for the comparison of basal with HVs or 0.967 for the comparison of attack with HVs (Supplementary Table S3, Supplementary Figure S5). Plasma HKa levels for HAE-C1INH patients in SCAT169 plasma collected between attacks were less differentiated from attack samples (AUC = 0.597).

ROC analysis of HKa ELISA data in citrated plasma also differentiated HAE-C1INH from HVs, with an AUC value of 0.915 for the comparison of basal with HVs or 0.866 for the comparison of attack with HVs (Supplementary Table S3, Supplementary Figure S5). HAE-C1INH basal citrated plasma samples were better differentiated from attack samples (AUC = 0.709) by an ELISA than by a WBA. ROC analysis suggests that the ELISA with SCAT169 plasma may be best suited for differentiating HAE-C1INH from HVs, as shown by ROC analysis, with an AUC value of 0.999 for the comparison of basal with HV or 1.0 for the comparison of attack with HV. Finally, the ELISA with SCAT169 plasma appeared best suited for the differentiation of HAE-C1INH basal from attack samples (AUC = 0.818).

HKa levels assessed by an ELISA in plasma collected using P100 tubes (BD Biosciences), which contain protease inhibitors, were lower than in citrated plasma from the same 30 individual HVs (Supplementary Figure S6). When the plasma was subjected to five freeze/thaw cycles, there was an apparent reduction in the ELISA signal after four cycles in citrated plasma and in P100 plasma collected in 8-ml tubes that contained a serum separator, but the ELISA signal appeared more stable in freeze/thaw cycles in P100 plasma collected in 2-ml tubes (Supplementary Figure S7).

**Figure 5:**
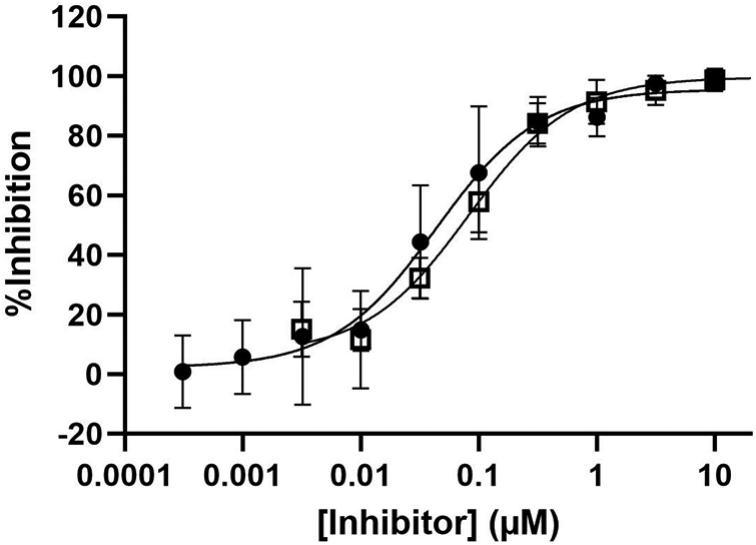
Inhibition of HKa generation in activated plasma by PKa inhibitors. Pooled sodium citrate human plasma samples were spiked with the antibody PKa inhibitor lanadelumab (closed circles) or a small-molecule PKa inhibitor BD-105294 (open squares). Plasma samples were activated for 30 min with 5 nM FXIIa. Post activation samples were analyzed at a 150-fold dilution. A dose-response relationship was fitted to averaged values from at least three experiments of duplicate measurements through non-linear regression (GraphPad Prism) using a three-parameter hyperbolic competitive equation {Y = Bottom + [Top-Bottom]/[1 + (X/IC_50_)]} to provide estimated IC_50_ values for lanadelumab [IC_50_ = 0.04 µM, with a 95% confidence interval (CI) from 0.030 to 0.064 µM] and BD-105294 (IC_50_ = 0.082 µM, with a 95% CI from 0.059 to 0.11 µM). FXIIa, coagulation factor XIIa; HKa, cleaved high-molecular-weight kininogen; IC_50_, half maximal inhibitory concentration; PKa, plasma kallikrein.

The complete inhibition of the HKa ELISA signal by the specific antibody inhibitor of PKa, lanadelumab (26, 27), with an IC_50_ of approximately 0.044 µM, or BD-105294, a novel small-molecule PKa inhibitor [the synthesis (28) of which is described in the supplementary methods] with an IC_50_ = 0.082 µM, indicates that the ELISA signal is PKa mediated (Figure 5). The observation that other reported PKa inhibitors were less effective in reducing HKa generation in activated plasma requires further investigation (Supplementary Table S4). However, confirmation that the HKa signal does indeed originate from PKa activity in activated plasma was obtained by multiple small-molecule PKa inhibitors screened at Takeda in the HKa ELISA and assessed for oral bioavailability in the rat (Supplementary Figure S9) and by EPI-KAL-2, a recombinant Kunitz domain PKa inhibitor previously discovered using phage display (29), which was also a potent inhibitor in the HKa ELISA with FXIIa activation (IC_50_ = 0.15 µM).

## 4 Discussion

The HKa-specific antibody M4-B4 was identified from a phage display library (19). The specificity of M4-B4 suggests that the antibody binds a neo-epitope(s) on HKa not present on HK or LK that is dependent on PKa or KLK1 activity. HK is a 626 amino acid glycoprotein that consists of six domains, and PKa or KLK1 cleave within domain four to release bradykinin or Lys-bradykinin, respectively, and generate HKa, which consists of two polypeptide chains linked by a disulfide (30). HKa has bioactivities (e.g., induction of endothelial cell apoptosis and inhibition of angiogenesis) not exhibited by HK (31), which is consistent with HKa having a distinct conformation from HK that is recognized by M4-B4. The increased binding affinity of M4-B4 for HKa in the presence of zinc suggests that the epitope may be near, or influenced by, the zinc-binding site on domain five of the light chain, which mediates the assembly of the KKS on cell surface receptors (32).

The importance of plasma collection methods and stabilization against proteolytic degradation has been described previously for the analysis of KKS activation (11). Contact of plasma with glass or other surfaces can result in extensive *ex vivo* KKS activation (6). Consequently, plasma obtained in this study relied on a blood collection protocol to minimize *ex vivo* KKS activation. *Ex vivo* KKS activation can be further minimized using an anticoagulant mixture that includes protease inhibitors (11, 13). In this study, *ex vivo* KKS activation, as measured by the concentration of HKa, was higher in citrated plasma from HVs and HAE-C1INH patients than in plasma collected in blood collection tubes that contained protease inhibitors (SCAT169 or P100 tubes).

The amount of HKa in citrated plasma by WBA during HAE-C1INH attacks was approximately 61.5% (Supplementary Table S1), which is similar to previous estimates (11, 33). An HKa ELISA in SCAT169 may provide estimates of the amount of HKa generated during an HAE-C1INH attack (4.4% or 32.7 nM), in basal HAE-C1INH conditions (2.2% or 16.2 nM), or in healthy controls (0.5% or 3.4 nM) (Supplementary Table S1). HKa concentrations should match the concentration of bradykinin generated during an attack, and the following evidence indicates that an approximately equivalent amount of PKa is generated during an HAE-C1INH attack: (1) the concentration of covalent PKa-α-macroglobulin complex (30–110 nM) during an attack (34) and (2) PKa estimates calculated from the extent of PK consumption during an attack (35, 36) and (3) from the maximum concentration (C_max_ = 83 nM) of ecallantide, which is a PKa inhibitor approved for treating an HAE-C1INH attack (37). Comparable concentrations of PKa and HKa suggest that when the KKS is activated at angioedema attack locations, the generated PKa cleaves HK with approximately equimolar stoichiometry (i.e., single turnover catalytic conditions).

Therefore, KKS activation occurring *in vivo* during an HAE-C1INH attack involves approximately equal concentrations of PKa protease and HK natural protein substrate and are expected to undergo fewer catalytic turnovers than *in vitro* reactions using a high concentrations of a synthetic peptide PKa substrate (e.g., 100 μM) that is at least 1000-fold higher than the concentration of PKa in undiluted activated plasma (∼100 nM) or in purified protein assays (∼1 nM). The higher catalytic turnovers expected with synthetic peptide substrates may contribute to the higher observed potency of certain reported PKa inhibitors measured using a fluorogenic synthetic peptide in minimally diluted plasma activated with FXIIa than with our HKa ELISA (see Supplementary Table S4). Furthermore, the use of 90% plasma in our HKa ELISA, as opposed to higher dilutions of plasma, may better predict endogenous PKa inhibition as the effects of interfering substances in the plasma (e.g., plasma protein binding) can be minimized at higher plasma dilutions and thereby artifactually increase the observed potency. The use of different KKS activators may also contribute to different observed potencies for PKa inhibitors; ellagic acid or dextran sulfate activates through inducing FXII autoactivation, the kinetics of which are not as immediate as directly adding FXIIa (38). Therefore, KKS activation induced by FXIIa addition may be expected to generate PKa more rapidly than other reported activators and thereby require higher inhibitor concentrations than KKS activators that act via FXII autoactivation. Consequently, our HKa ELISA may be a useful *in vitro* assay for helping to predict *in vivo* efficacy for PKa inhibitors in development for HAE-C1INH and other indications potentially mediated by PKa, such as bradykinin-mediated angioedema (39), diabetic macular edema (40), Alzheimer's disease (15), ischemic stroke (41), myocardial infarction (42), systemic lupus erythematosus (43), cancer (44), and sepsis (45).

## Data Availability

The original contributions presented in the study are included in the article/Supplementary Material, further inquiries can be directed to the corresponding author.
