# Corrigendum: A novel assay of excess plasma kallikrein-kinin system activation in hereditary angioedema

**DOI:** 10.3389/falgy.2025.1557356

**Published:** 2025-02-14

**Authors:** Dan Sexton, Ryan Faucette, Melody Rivera-Hernandez, Jon A. Kenniston, Nikolaos Papaioannou, Janja Cosic, Kris Kopacz, Gary Salmon, Chantal Beauchemin, Salomé Juethner, Dave Yeung

**Affiliations:** ^1^ Takeda Development Center Americas Inc., Cambridge, MA, United States; ^2^ Charles River Laboratories, Harlow, United Kingdom; ^3^ Takeda Pharmaceuticals USA, Inc., Lexington, MA, United States

**Keywords:** biomarkers, phage display, bradykinin, plasma kallikrein, hereditary angioedema

A corrigendum on A novel assay of excess plasma kallikrein-kinin system activation in hereditary angioedema By Sexton D, Faucette R, Rivera-Hernandez M, Kenniston JA, Papaioannou N, Cosic J, Kopacz K, Salmon G, Beauchemin C, Juethner S and Yeung D (2024). Front. Allergy. 5:1436855. doi: 10.3389/falgy.2024.1436855

**Incorrect Copyright Statement**

In the published article, there was an error in the Copyright statement. The statement was incorrectly written as “Copyright **©** 2024 Sexton, Faucette, Rivera-Hernandez, Kenniston, Papaioannou, Cosic, Kopacz, Salmon, Beauchemin, Juethner and Yeung”. The corrected statement is “This work is authored by Sexton, Faucette, Rivera-Hernandez, Kenniston, Papaioannou, Cosic, Kopacz, Salmon, Beauchemin, Juethner, and Yeung. © 2024 Takeda Pharmaceuticals USA Inc. for Sexton, Faucette, Rivera-Hernandez, Kenniston, Papaioannou, Cosic, Kopacz, Salmon, Beauchemin, Juethner, and Yeung”. This is an open-access article distributed under the terms of the Creative Commons Attribution License (CC BY). The use, distribution or reproduction in other forums is permitted, provided the original authors and the copyright owner are credited and the original publication in this journal is cited, in accordance with accepted academic practice. No use, distribution or reproduction is permitted which does not comply with these terms.”

The authors apologize for this error and state that this does not change the scientific conclusions of the article in any way. The original article has been updated.

