# Erratum: A novel assay of excess plasma kallikrein-kinin system activation in hereditary angioedema

**DOI:** 10.3389/falgy.2025.1608925

**Published:** 2025-04-25

**Authors:** Frontiers Production Office

**Affiliations:** Frontiers Media SA, Lausanne, Switzerland

**Keywords:** biomarkers, phage display, bradykinin, plasma kallikrein, hereditary angioedema

An erratum on A novel assay of excess plasma kallikrein-kinin system activation in hereditary angioedema Sexton D, Faucette R, Rivera-Hernandez M, Kenniston JA, Papaioannou N, Cosic J, Kopacz K, Salmon G, Beauchemin C, Juethner S and Yeung D (2024). Front. Allergy 5:1436855. doi: 10.3389/falgy.2024.1436855

Due to a production error, Supplemental material Data sheet 1 was omitted. The file has now been published.

The publisher apologizes for this mistake.

The original version of this article has been updated.

