# Supplementary material for: A novel assay of excess plasma kallikrein-kinin system activation in hereditary angioedema
Source: Front Allergy. 2024 Sep 17;5:1436855. doi: 10.3389/falgy.2024.1436855 (PMC11464748; doi:10.3389/falgy.2024.1436855)
Supplement: Supplementary file 1 [file Datasheet1.docx]

Supplementary Material

**Supplementary Figures**

**Supplementary Figure 1.** Comparison of M4-B4 (aka M004-B04) specificity with two other antibodies identified by phage display. HK, high-molecular-weight kininogen; HKa, cleaved high-molecular-weight kininogen; LK, low-molecular-weight kininogen; OD, optical density.

**Supplementary Figure 2.** The addition of zinc chloride (ZnCl_2_) increases M4-B4 ELISA signal in ellagic acid treated citrated or EDTA human plasma.


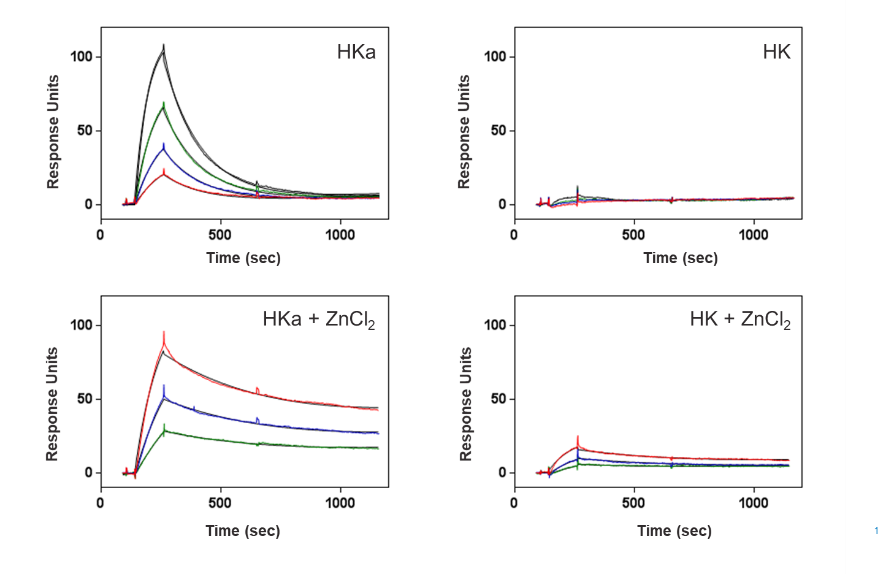


**Supplementary Figure 3.** Surface plasmon resonance analysis of HKa or HK binding to M4-B4 in the presence or absence of zinc chloride (ZnCl_2_). Measurement of binding kinetics of M4-B4 to different forms of kininogen were performed using a Biacore 3000 instrument (GE Healthcare Life Sciences) with the detection temperature at 25°C and HBS-P running buffer (10 mM HEPES, pH 7.4, 150 mM sodium chloride, and 0.005% surfactant P20) with and without 1M EDTA or 200 µM ZnCl_2_. The different forms of kininogen (intact HK and HKa) were immobilized by amine-coupling on a CM5 sensor chip at ~400 response units, and M4-B4 injected for 2 minutes at 50 μL/min followed by a 15-minute dissociation phase. Surfaces for experiments with included ZnCl_2_ were regenerated with a 32-second pulse of 10 mM glycine (pH 1.5) at 75 μL/min. Kinetic association (k_on_) and dissociation (k_off_) constants were obtained by using the Biaevaluation software with the model for the formation of a complex with a 1:1 stoichiometry. HK, high-molecular-weight kininogen; HKa, cleaved high-molecular-weight kininogen.

**Supplementary Figure 4.** Cleaved high-molecular-weight kininogen (HKa) ELISA standard curve. OD, optical density. The standard curve was fit by nonlinear regression analysis to the four parameter logistic equation: $y=d+(a-d)/(1+\left( \frac{x}{c} \right)^{b})$ where y = OD, a = the minimum value that can be obtained, d = the maximum value that can be obtained, c = the point of inflection, and b = Hill’s slope of the curve.

**Supplementary Figure 5.** ROC curves for plasma HKa in HVs and HAE-C1INH patients. The area under the curve (AUC or C-statistic) is indicated on each curve.

**Supplementary Figure 6.** Comparison of cleaved high-molecular-weight kininogen (HKa) levels in healthy volunteers by M4-B4 enzyme-linked immunosorbent assay (ELISA) in citrated plasma versus plasma collected in P100 tubes (either 8 mL or 2 mL total volumes from BD Biosciences) from 30 individual healthy volunteers. Coat Nunc MaxiSorp plates with 3 µg/mL of M4-B4 capture antibody in 0.2 M carbonate bicarbonate and let incubate at 4°C, shaking, overnight. On the day of the assay, wash coated plates with a solution of 1× PBS and 0.05% Tween 20 at least 3 times. Blot plates to remove as much of the washing buffer as possible and block plates with 300 µL of 1× PBS, 5% BSA and 0.05% Blocking Reagent solution and let it incubate for 1 hour, shaking at RT. During the incubation period, samples, standard curves and controls can be prepared. Dilute reference standard, HKa in sample dilution buffer (1× PBS, 1% BSA, 0.01% Blocking Reagent) at a starting concentration of 250 ng/mL, proceeding with 10 more 1:2 serial dilutions and ending the standard curve with a dilution buffer blank. All plasma samples were diluted and 1:300, 1:600, and 1:1200. After an hour of blocking, plates can be washed again using the process described above and 100 µL of samples, standards and controls can be added to the plate. Immediately after an hour of sample incubation, plates are washed and 0.5 µg/mL of 11H05 detection antibody is added to each well and incubated for 1 hour, shaking at RT. In order to detect a signal, a 1:100,000 solution of Goat anti-mouse IgG-HRP secondary antibody needs to be added after the detection step and incubated for another hour. Once the ELISA is complete, the plate is washed and 50 µL of TMB Substrate solution is added to each well. After 10–15 minutes of color development, 100 µL of Sulfuric Acid 2N is added to each well to stop the reaction. The absorbance is read immediately in a SpectraMax microplate reader at 450 nm with a 630-nm correction. HRP, horseradish peroxidase.

**Supplementary Figure 7.** Comparison of HKa levels in plasma after multiple freeze thaws by M4-B4 ELISA in citrated plasma versus plasma collected in P100 tubes (either 8 mL or 2 mL total volumes from BD Biosciences) from 30 individual healthy volunteers. NaCit, sodium citrate; HKa, cleaved high-molecular-weight kininogen.

559B-M0004-B04 HC IgG

EVQLLESGGGLVQPGGSLRLSCAASGFTFSFYVMVWVRQAPGKGLEWVSGISPSGGNTAYADSVKGRFTISRDNSKNTLYLQMNSLRAEDTAVYYCARKLFYYDDTKGYFDFWGQGTLVTVSSASTKGPSVFPLAPSSKSTSGGTAALGCLVKDYFPEPVTVSWNSGALTSGVHTFPAVLQSSGLYSLSSVVTVPSSSLGTQTYICNVNHKPSNTKVDKRVEPKSCDKTHTCPPCPAPELLGGPSVFLFPPKPKDTLMISRTPEVTCVVVDVSHEDPEVKFNWYVDGVEVHNAKTKPREEQYNSTYRVVSVLTVLHQDWLNGKEYKCKVSNKALPAPIEKTISKAKGQPREPQVYTLPPSREEMTKNQVSLTCLVKGFYPSDIAVEWESNGQPENNYKTTPPVLDSDGSFFLYSKLTVDKSRWQQGNVFSCSVMHEALHNHYTQKSLSLSPG

559B-M0004-B04 LC (Lambda)MW

QYELTQPPSASGTPGQRVTLSCSGSSSNIGSNYVYWYQQLPGTAPKLLIYRNNQRPSGVPDRFSGSKSGTSASLAISGLQSEDEADYYCAAWDDSLNGRVFGGGTKLTVLGQPKAAPSVTLFPPSSEELQANKATLVCLISDFYPGAVTVAWKADSSPVKAGVETTTPSKQSNNKYAASSYLSLTPEQWKSHRSYSCQVTHEGSTVEKTVAPTECS

**Supplementary Figure 8.** M4-B4 amino acid sequence.


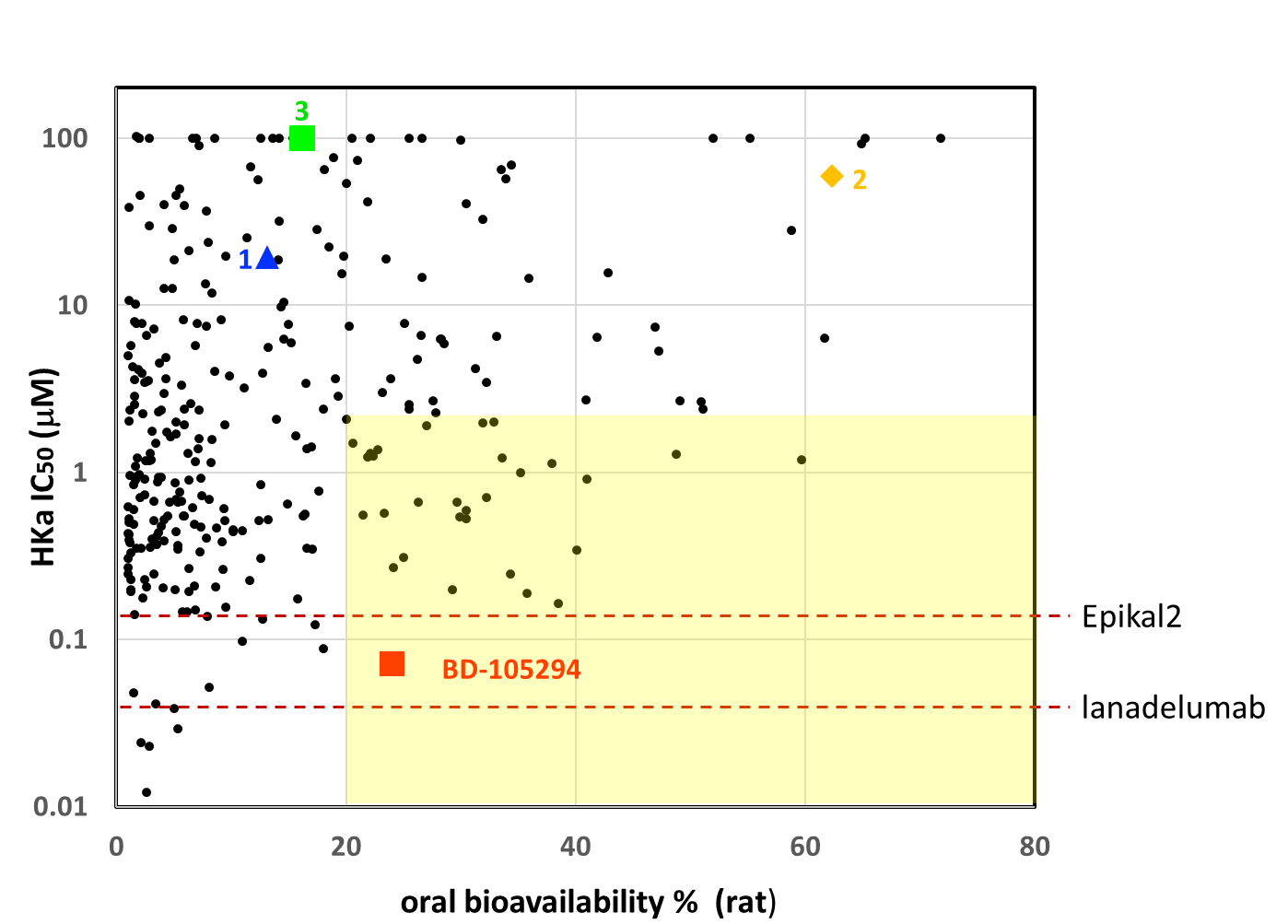


Supplementary Figure 9. Comparison of PKa inhibitors potencies versus oral bioavailability in rat. Pharmacokinetic assessment of small-molecule plasma kallikrein inhibitors were performed with Sprague Dawley male rats. Pharmacokinetic studies were performed by intravenous (IV) injection and oral (PO) gavage. Three animals were included in each dose group for PO and IV studies. Blood sampling was performed at 9 times points for IV and 8 times point for PO administration. Previously reported PKa inhibitors 1, 2, and 3 in Supplementary Table 4 are highlighted along with a previously unreported PKa small molecule inhibitor BD-105294. The IC_50_ of EPI-KAL2 and lanadelumab are indicated by the red dashed lines and as both are biologic PKa inhibitors were not tested for oral bioavailability.

**Supplementary Tables**

**Supplementary Table 1.**

| Plasma HKa levels in HVs and patients with HAE-C1INH | **HKa Western blot** | | | | | |
| --- | --- | --- | --- | --- | --- | --- |
|  | Citrated plasma (Figure 4A) | | | SCAT169 plasma (Figure 4B) | | |
|  | HV | Basal | Attack | HV | Basal | Attack |
| N | 52 | 55 | 20 | 26 | 105 | 46 |
| Minimum (%HKa) | 2.9 | 6.2 | 31.91 | 3.2 | 2.6 | 5.8 |
| Maximum (%HKa) | 29.5 | 100 | 100 | 12.7 | 81 | 100 |
| Median (%HKa) | 9.4 | 49.5 | 62.3 | 6.6 | 19.9 | 24.2 |
| Mean (%HKa) | 10.2 | 51.4 | 61.5 | 6.6 | 21.6 | 26.4 |
| SD (%HKa) | 4.9 | 23 | 23.8 | 2 | 13 | 16.8 |
|  | **HKa ELISA** | | | | | |
|  | [HKa] in citrated plasma (Figure 4C) | | | [HKa] in SCAT169 plasma (Figure 4D) | | |
|  | HV | Basal | Attack | HV | Basal | Attack |
| N | 24 | 14 | 14 | 39 | 17 | 10 |
| Minimum (ng/mL) | 325.9 | 1831 | 1631 | 215.1 | 523.7 | 1645 |
| Maximum (ng/mL) | 18641 | 13057 | 30776 | 540.4 | 5189 | 8758 |
| Median (ng/mL) | 1111 | 3833 | 5304 | 357.4 | 1299 | 3167 |
| Mean (ng/mL) | 2966 | 4994 | 9389 | 370.7 | 1780 | 3599 |
| Mean (%HKa)^a^ | 3.6 | 6.1 | 11.5 | 0.5 | 2.2 | 4.4 |
| Mean (nM)^b^ | 27.0 | 45.4 | 85.4 | 3.4 | 16.2 | 32.7 |
| SD (ng/mL) | 4263 | 3288 | 8023 | 91.83 | 1246 | 2219 |
| ^a^ Expressed as a percent of total HK 82,000 ng/mL [Scott CF, Shull B, Muller-Esterl W, Colman RW. Rapid direct determination of low and high-molecular-weight kininogen in human plasma by particle concentration fluorescence immunoassay (PCFIA). *Thromb Haemost.* 1997;77:109-118.] | | | | | | |
| ^b^ Calculated using a HK molecular weight of 110 kDa. | | | | | |  |

HAE-C1INH, hereditary angioedema due to a deficiency in total (type I) or functional C1 inhibitor protein (type II); HK, high-molecular-weight kininogen; HKa, cleaved high-molecular-weight kininogen; HV, healthy volunteer; SD, standard deviation.

**Supplementary Table 2.** Kinetic constants obtained from surface plasmon resonance analysis of HKa or HK binding to M4-B4 in the presence or absence of ZnCl_2_

|  |  | **k_a_ (1/Ms)** | **k_d_ (1/s)** | **K_D_ (M)** | **Chi_2_** |
| --- | --- | --- | --- | --- | --- |
| No added ZnCl_2_ | Replicate 1 | 1.14E+06 | 6.70E-03 | 5.90E-09 | 0.254 |
|  | Replicate 2 | 9.84E+05 | 6.16E-03 | 6.26E-09 | 0.828 |
|  | Average | 1.06E+06 | 6.43E-03 | 6.08E-09 | 0.541 |
|  | SD | 1.10E+05 | 3.82E-04 | 2.55E-10 |  |
| With 200 µM ZnCl_2_ | Replicate 1 | 1.15E+06 | 1.25E-03 | 1.09E-09 | 1.56 |
|  | Replicate 2 | 1.38E+06 | 1.72E-03 | 1.24E-09 | 0.852 |
|  | Replicate 3 | 1.77E+06 | 1.56E-03 | 8.81E-10 | 0.27 |
|  | Average | 1.58E+06 | 1.64E-03 | 1.06E-09 | 0.894 |
|  | SD | 2.76E+05 | 1.13E-04 | 2.54E-10 |  |

HK, high-molecular-weight kininogen; HKa, cleaved high-molecular-weight kininogen; SD, standard deviation; ZnCl_2_, zinc chloride.

**Supplementary Table 3.** Comparison between plasma HKa levels between HVs and HAE-C1INH patients

|  |  | HV vs basal | HV vs attack | Basal vs attack |
| --- | --- | --- | --- | --- |
| %HKa in citrated plasma by Western blot | Mann-Whitney test | *P*<0.0001 | *P*<0.0001 | *P*=0.1022 |
|  | ROC C-statistic | 0.9773 | 1.00 | 0.6245 |
|  | Difference between mean (%HKa) | 41.2 | 51.3 | 10.1 |
| %HKa in SCAT169 plasma by Western blot | Mann-Whitney test | *P*<0.0001 | *P*<0.0001 | *P*=0.0579 |
|  | ROC C-statistic | 0.9147 | 0.9666 | 0.5971 |
|  | Difference between mean (%HKa) | 15 | 19.8 | 4.8 |
| HKa in citrated plasma by ELISA | Mann-Whitney test | *P*=0.0021 | *P*<0.0001 | *P*=0.0062 |
|  | ROC C-statistic | 0.7946 | 0.8661 | 0.7092 |
|  | Difference between mean (ng/mL) | 2232 | 4052 | 1471 |
| HKa in SCAT169 plasma by ELISA | Mann-Whitney test | *P*<0.0001 | *P*<0.0001 | *P*=0.0056 |
|  | ROC C-statistic | 0.9985 | 1.00 | 0.8176 |
|  | Difference between means (ng/mL) | 941.6 | 2810 | 1868 |

HAE-C1INH, hereditary angioedema due to a deficiency in total (type I) or functional C1 inhibitor protein (type II); HKa, cleaved high-molecular-weight kininogen; HV, healthy volunteer; ROC, receiver operator characteristic.

**Supplementary Table 4.** Comparison of PKa inhibitor potency

| PKa inhibitor | Ki (nM) or IC_50_ (nM) ^a^ | HKa ELISA IC_50_ (nM)^h^ | Fluorogenic peptide IC_50_ (nM)^i^ |
| --- | --- | --- | --- |
| Lanadelumab | 0.12 ^b^ | 40 | 22 |
| EPI-KAL2 (ecallantide surrogate) | 0.1 c | 150 | 55 |
| PKa Inhibitor 1 | 0.9^d^ | 60,000 | 34 |
| PKa Inhibitor 2 | 6 (IC_50_)^e^ | >100,000 | 124 |
| PKa Inhibitor 3 | 2.7 (IC_50_)^f^ | >100,000 | 275 |
| BD-105294 | 0.11 (IC_50_)^g^ | 82 | 57 |

^a^Ki (inhibition constant) or half maximal concentration (IC_50_) values were collated from literature or internal measurements.

^b^Kenniston JA, et al. *J Biol Chem*. (2014) 289, 23596-608.

^c^Markland W, et al. *Biochemistry.* (1996) 35, 8058-67.

Kotian PL, et al*. J Med Chem*. (2021) 64, 12453-12468.

^e^Davie RL, et al. *J Med Chem*. (2022) 65, 13629-44.

^f^Kalfus I, et al. *J Allergy Clin Immunol.* (2017) 139(2):AB378.

^g^IC_50_ value generated internally using purified enzymes similar to the method reported in Kenniston JA, et al. *J Biol Chem*. (2014) 289(34):23596-608.

^h^IC_50_ values determined using the cleaved high-molecular-weight kininogen (HKa) ELISA described here with FXIIa activation within 90% human plasma.

^i^IC_50_ values determined using 78% human plasma activated with FXIIa and monitored plasma kallikrein (PKa) activity using the synthetic fluorescent substrate Pro-Phe-Arg-aminomethylcoumarin (100 μM).

**Supplementary Methods**

**Synthesis of BD-105294: 1-((6-cyclopropylimidazo[1,2-b]pyridazin-2-yl)methyl)-N-(2-fluoro-3-methoxy-6-(1H-tetrazol-1-yl)benzyl)-1H-1,2,3-triazole-4-carboxamide**

To a solution of (2-fluoro-3-methoxy-6-(1H-tetrazol-1-yl)phenyl)methanamine^1^ (4.4 g, 19.7 mmol) in dry DMF (50 mL) was added 1-((6-cyclopropylimidazo[1,2-b]pyridazin-2-yl)methyl)-1H-1,2,3-triazole-4-carboxylic acid^2^ (4.6 g, 16.4 mmol), HOBT (2.9 g, 21.6 mmol), EDCI (4.1 g, 21.6 mmol) and DIPEA (6.3 g, 49.2 mmol). The reaction was stirred at room temperature for 16 h. After the reaction was completed, the solution was poured into water (500 mL) slowly, and the white solid was precipitated. The solid was filtered, the filtered cake was washed with H_2_O (300 mL) and dried to give crude, which was triturated with (DCM / MeOH = 20 / 1, 50 mL) to give 1-((6-cyclopropylimidazo[1,2-b]pyridazin-2-yl)methyl)-N-(2-fluoro-3-methoxy-6-(1H-tetrazol-1-yl)benzyl)-1H-1,2,3-triazole-4-carboxamide (4.6 g, yield: 57.5%). ESI-MS [M +H]+: 490.1. Purity: 99.5 %. 1H NMR (400 MHz, DMSO) δ 9.74 (s, 1H), 8.75 (t, J = 5.2 Hz, 1H), 8.50 (s, 1H), 8.18 (s, 1H), 7.92 (d, J = 9.4 Hz, 1H), 7.39 – 7.32 (m, 2H), 7.10 (d, J = 9.5 Hz, 1H), 5.73 (s, 2H), 4.29 (d, J = 5.0 Hz, 2H), 3.92 (s, 3H), 2.20 – 2.14 (m,, 1H), 1.08 – 1.04 (m, 2H), 0.98 – 0.96 (m, 2H).

1. Davie, R.L; Edwards, H.J; Evans, D.M.; Hodgson, S.T.; Pethen, S.J; Rooker, D.P. Enzyme Inhibitors. US202/0275 A1, January 6, 2022.

2. Papaioannou, N.; Fink, S.J.; Miller, T.A.; Shipps, G.W.; Travins, J.M.; Ehmann, D.E.; Rae, A; Ellard, J.M. Inhibitors of Plasma Kallikrein and Uses Thereof. US 10730874 B2, August 4, 2020.
